# Synergistic activity of sunlight protectants on some biocontrol agents as a new approach to control the cotton leaf worm, *Spodoptera littoralis* (Boisduval)

**DOI:** 10.1038/s41598-026-35601-7

**Published:** 2026-02-03

**Authors:** Radwa G. Attia, Abd El Aziz A. Khidr, Hend A. A. Al-Ashry, M. S. Yones, Mahmoud Sayed, Shireen A. M. Maamoun

**Affiliations:** 1 Entomology Department, Faculty of Science, Ain Shams University, Cairo, Egypt; 2 Plant Protection Research Institute, Agricultural Research Center, Dokki, Giza Egypt; 3 National Authority for Remote Sensing and Space Sciences, Cairo, Egypt

**Keywords:** Ultraviolet protectant, Bioinsecticides, Spodoptera littoralis, Total protein, Biological techniques, Ecology, Ecology, Zoology

## Abstract

Bioinsecticides have gained attention as a sustainable and eco-friendly alternative to traditional chemical insecticides. They are target-specific, biodegradable, and reduce the development of insect resistance. However, their use is often limited by environmental instability, particularly under sunlight and UV radiation, which rapidly degrade active ingredients and reduce efficacy. Protective formulation, such as addition of UV-screening agents are needed to improve their durability and efficacy. This study investigates the efficiency of three UV protectants, Octylpalmitate, Tinuvin P, and UV- P in enhancing the persistence of some biocontrol agents, Dipel DF, Tracer, and Diacox under field conditions for 15 days to control the 4th instar larvae of *Spodoptera littoralis*. After 12 days, the residual effect of the all insecticides had markedly decreased, with Tracer showing the lowest persistence with zero mortality, and Diacox + UV protectant the highest. By 15 days, the sustained efficacy was absent, except for Diacox mixed with Octyl palmitate, Tinuvin P, and UV-P, which still caused 27, 32 and 12% mortality; respectively. The SDS-PAGE of *S. littoralis* larvae following 15 days of treatments revealed differences in the numbers of protein fragments between treated and control. New protein bands formed in the treated samples, while several normal bands disappeared.

## Introduction

The Egyptian cotton leaf worm, *Spodoptera littoralis* (Boisd.), is considered as one of the most important and damaging pests in Egypt. Larvae cause severe damage in numerous kinds of cultivated crops, particularly cotton, resulting in substantial economic loss through the consumption of, both plant, vegetative and fruiting structures^1^. Chemical insecticides are widely used to control cotton leaf worm. Repeated use of such insecticides led to developing of resistance in insect population. They also cause environmental pollution and disrupts ecological balance between the pest and its natural enemies. Modern pest control highlights integrated pest management that combine biological, cultural, and chemical methods to reduce reliance on chemical insecticides. Hence, it is necessary to implement a new strategy that integrates economic and pest control data to convince farmers.

Recently, there are many compounds that exhibit novel mode of actions, from these compounds, the bio control agent, Emamectin benzoate (methyl amine avermectin). It is considered the second generation of abamectin family and acts as a nerve poison. It is stored as a reserve in the plant’s parenchyma tissue, where it functions as a defense mechanism against phytophagous insects^1^. Its main physiological mechanism of action is to induce the release of gamma-amino butyric acid (GABA) neurotransmitter, leading to a continued inflow of chloride ions into muscles cells, which results in suppressed contraction and paralysis^2^. The second product, the entomopathogenic bacteria, Dipel 6.4% DF (*Bacillus thringiensis* subsp. *Kurstaki*), the most widely used microbial insecticide and have been known to be highly toxic to certain insects with no reported adverse effect to human, beneficial insects and other target organisms. The third bioinsecticide exhibited moderate to high acute toxicity to insect species is Spinosad, which considered the first active component suggested for a novel class of insect control products^3^. It has been demonstrated that Spinosad is a naturally derived biocide originated from filamentous, non-motile, gram-positive bacteria, *Saccharopolyspora spinosa* and made up of two macrocyclic lactones, spinosyn A and spinosyn D, and shown to be effective against insects, including those belonging to the order Lepidoptera, Diptera, Hymenoptera, Thysanoptera and few Cleoptera^4^. Spinosad degrades rapidly in the environment and has low persistence^5^.

It was observed that bioinsecticides gradually lose their original potency after exposure to ultraviolet radiation (UV) under field conditions^6,7^. Thus persistence and efficacy of those bioinsecticides could be reinforced by using UV protectants in order to accomplish their role as selective and efficient larvicides of cotton pests. Sunscreen active ingredients are classified by the Food and Drug Administration (FDA) as substances that absorb, reflect, or scatter UV radiation with wavelengths between 290 and 400 nanometers. Benzotriazole ultraviolet light absorbers, such as UV-P and Tinuvin P (2-(2 H-benzotriazol-2-yl)-p-cresol), protect coatings, plastics and other organic materials from the harmful effects of UV radiation by absorbing possibly harmful UV radiation and disperse the energy as heat. They prevent material deterioration and discoloration by absorbing UV radiation in the range of 300–400 nm^8^. Octyl palmitate (C_24_H_48_O_2_) serves as an emollient, dispersant and solvent in cosmetics and sunscreens^9^. It enhances the feel and smoothness of the skin and improves the effectiveness of sunscreen products by acting as a vehicle for the actual UV-blocking substances. **El-Husseini et al.**^10^ established that the addition of some natural products like clay, glycerin, black tea extract and starch as UV protectant improved the persistence of *S*. *littoralis* nucleopolyhedrovirus under field condition, leading to a marked increase in larval mortality. Despite the fact that few studies have assessed the potential of UV protectants to increase the effectiveness and durability of bioinsecticides in field applications, all investigations indicated that the protection of bioinsecticides is crucial to preserving their efficacy under field conditions. Exposure of unprotected bioinsecticides to UV radiation for several hours could affect their binary toxin protein bands. This may be due to the generation of peroxide radicals from UV radiation, which results in the loss bioinsecticides activity^11^.

The present study targets to assess the suitability of some UV protectants in shielding bioinsecticides from degradation by UV radiation. The residual activity of applied UV-protected bioinsecticides on leaves of cotton plants was evaluated to control the 4th instar larvae of *S. littoralis*. A qualitative protein analysis was also performed to validate the suggested bioactivity enhancement results.

## Materials and methods

### Insect tested

A laboratory strain of the cotton leaf worm, *Spodoptera littoralis* (Boisd.), was acquired from Cotton Leaf worm Research Department, Plant Protection Research Institute, Dokki, Giza, Egypt. A pure strain was reared for more than 12 generations at 27 ± 1 °C and 65 ± 5% R.H under laboratory conditions without any insecticide contamination. Fresh castor bean leaves were provided to the standard tested insect used in the bioassay of the experiments every day according the method described by **Khidr et al.**^12^.

### Biocides used

The biocides used, active ingredients, chemical or biological class, and recommended rate are listed in Table 1.

**Table 1:** Trade name, active ingredient and class type and recommended rate of the biocides used.

Biocides used	Trade name	Active ingredient	Chemical or biological class	Recommended rate
*Bacillus thuringiensis var. Kurstaki*.	Dipel 6.4% DF	*Bacillus thuringiensis var. kurstaki* (Btk)	Bioinsecticide derived from soil bacterium, *Bacillus thuringiensis*	476 g/ha.
Spinosad	Tracer 24% SC	Spinosyn A and Spinosyn D	Spinosyns(Biologically derived from *Saccharopolyspora spinosa*)	119 ml/ha.
Emamectin benzoate	Diacox 5.7% WG	*Streptomyces avermitilis*	Avermectin (Biologically derived from soil bacterium, *Streptomyces avermitilis*)	143 g/ha.

### Ultraviolet protectant

Octyl pulmitate at recommended rate of 47.6 g/ha.

Tinuvin P at recommended rate of 47.6 g/ha.

UV-P at rate recommended of 47.6 g/ha.

### Preparation of biocontrol agents used alone

Each of biocontrol agents; 10 g Dipel 6.4% DF, 2.5 ml tracer and 3 g Diacox was added to 10 L water. Each was prepared for testing their efficacy alone under field conditions.

### Treatments

Each of 10 g Dipel 6.4%DF, 2.5 ml Tracer and 3 g Diacox was mixed with one gram of each ultraviolet protectant; Octylpulmitate, Tinuvin P, and UV-P, dissolved in 10 L water, as following:

Dipel 6.4%DF + Octyl palmitate (10: 1); Dipel 6.4%DF + Tinuvin P (10:1); Dipel DF + UV-P (10:1).

Tracer + Octyl palmitate (2.5:1); Tracer + Tinuvin P (2.5:1); Tracer + UV-P (2.5:1).

Diacox + Octyl palmitate (3:1); Diacox + Tinuvin P (3:1); Diacox + UV-P (3:1).

### Field-laboratory experiments

According to randomized block design, the experimental area (1/2 feddan) at Qaha Research Station, Qalubia Governorate was divided into 13 plots. Seeds of cotton variety Giza 89 were cultivated in 12 plots for different treatments and one plot for control. The insecticidal treatments as well as untreated check plots (180 square meters of each) were distributed randomly between plots. The average temperature ranged between 33 and 35 °C, with a relative humidity of 60–62%, and an average wind speed of 3–3.4 m**/s**. Knapsack sprayer capacity 25 L was used for the insecticidal application. The insecticidal application was implemented in the field on August, 21st during the growing cotton season, 2022. Untreated check plot was sprayed with water only.

The three biocontrol agents, Dipel DF6.4%, tracer and Diacox were prepared and applied separately. Also, the mixed form of these tested biocontrol agents with each of the three ultraviolet (UV) protectants were prepared. Each treatment was sprayed once, on the cotton leaves, in its assigned check plot.

### Determination of the persistence of the tested treatments under field conditions

Samples of 50 cotton, treated and untreated, leaves were collected from each plot in the field after 0, 3, 6, 9, 12 and 15 days from sunlight exposure. They were kept in plastic bags, then transferred to the laboratory of Cotton Leaf Worm Research Department, Plant Protection Research Institute. The experiments were conducted under laboratory conditions of 25 ± 2 °C, 70 ± 5% RH. Ninety individuals of the 4th instar larvae were divided into three replicates (30 larvae / replicate). Each replicate was placed in a glass jar (15 cm in diameter X 30 cm in high) and exposed to the treated leaves. Whereas, the larvae of the control group were exposed to untreated cotton leaves (sprayed with water only). The evaluation of the insecticidal efficacy was based on the mortality percentages after three days interval from each tested period. Samples were inspected at 0, 3, 6, 9, 12 and 15 days for each treatment. Average of mortality percentages were corrected using **Abbott’s formula**^13^. The toxicity index **(Sun)**^14^ and potency levels were also calculated.

$${\mathrm{Sun}}{\prime }{\text{s Toxicity index}}=\frac{{{\text{The mean percent mortality of the tested treatments}}}}{{{\text{The highest mean percent mortality of the standard}}}} \times 100$$

$${\text{Potency levels}}=\frac{{{\text{The mean percent mortality of the tested treatments}}}}{{{\text{The lowest mean percent mortality of the standard}}}}$$

### Qualitative analysis of total protein

Total protein of untreated 4th instar larvae of *S. littoralis* (control) as well as treated larvae with the three bioinsecticides alone and after addition of UV protectants were separated using sodium dodecyl sulfate polyacrylamide gel electrophoresis (SDS-PAGE) according to **Laemmli**^15^. Total protein was carried out on larvae treated with collected leaves after 15 days of sunlight exposure. Following electrophoresis, the entire protein gel was submerged in Coomassie Brilliant Blue R-250 staining solution for 12 to 18 h, followed by a destaining solution (methanol and glacial acetic acid) to view the bands. The protein pattern was analyzed by using Syngene gene tools software, version (4.3.14.0).

### Statistical analysis

Computer statistical software (SPSS) version 27.0 was used for comparison between the mean mortality percentage in all days of treatments. One-way analysis of variance (ANOVA) was conducted on the gathered data, followed by least significant difference (LSD) test for pairwise comparison at p value < 0.05 to determine significant differences between treatments. To compare the efficacy of the tested bioinsecticides and UV protectants, a binomial generalized linear model (GLM) was performed. Treatment effects were evaluated using Wald’s chi-square test, and significance was determined at *p* < 0.001.

## Results

The persistence of an insecticide toxicity depends on its chemical properties, biology of the target pest, the duration of the growing season of the used crop, and the impact of environmental factors, particularly ultraviolet light-induced degradation. The efficacy of three ultraviolet (UV) protectants; Octyl Palmitate, Tinuvin P, and UV-P was investigated for their ability to enhance the residual activity of bioinsecticides against 4th instar larvae of the cotton leafworm, *Spodoptera littoralis*. The treatments were compared at 0, 3, 6, 9, 12, and 15 days following application to assess their effectiveness over time. The results represented the initial mortality percentages and persistence of the different treatments (Table 2, 4, 6).

**Table 2:** Potency of the ultraviolet protectant, octyl palmitate for increasing the toxicity of three bioinsecticides against the 4th instar larvae of *S. littoralis* under field-laboratory conditions.

Compounds used	Rate/Plot	%Mean mortality ± SE						%Mean mortality	Improve ratio^*^	Toxicity Index	Relative potency levels
		Initial kill 0-day	Days after treatment								
			3	6	9	12	15				
Dipel DF	10 g	84 ± 1.73^a^	72 ± 2.31^a^	42 ± 2.31^a^	15 ± 2.31^a^	3 ± 0.58^a^	0.00 ± 0.00^a^	36.0	–	47.57	1.16
Dipel DF + Octyl palmitate	10 g + 1 g	90 ± 1.73^b^	81 ± 1.73^b^	63 ± 1.73^b^	42 ± 3.46^b^	21 ± 1.73^b^	0.00 ± 0.00^a^	49.50	1.38	65.41	1.60
Tracer	2.5 g	81 ± 16^a^	66 ± 1.73^c^	30 ± 1.16^c^	9 ± 0.00^a^	0 ± 0.00^c^	0.00 ± 0.00^a^	31.0	–	40.97	1.00
Tracer + Octyl palmitate	2.5 ml + 1 g	81 ± 2.31^a^	81 ± 1.61^b^	66 ± 2.89^b^	36 ± 1.73^bc^	24 ± 0.58^d^	0.00 ± 0.00^a^	48.0	1.55	63.43	1.55
Diacox	3 g	100 ± 0.00^c^	96 ± 2.31^d^	69 ± 2.89^b^	30 ± 1.16^c^	9 ± 0.58^e^	0.00 ± 0.00^a^	50.67	–	66.96	1.63
Diacox + Octyl palmitate	3 g + 1 g	100 ± 0.00^c^	96 ± 1.73^d^	90 ± 2.31^d^	81 ± 2.89^d^	60 ± 1.16^f^	27 ± 0.58^b^	75.67	1.49	100	2.44

Different letters within the same column indicate statistical difference at the 0.05 level.

The toxicity of the three biocides either implemented alone or combined with the UV protectant, Octyl palmitate was assessed. The experimental results revealed that the first mortality of the tested compounds was observed at 0-day as well as their persistence after three days interval from spraying. The mortality percentages ranged between 81 and 100% (Table 2). After that the persistence of the six treatments declined gradually at 3, 6 and 9 days after treatment. Based on persistence, the highest mortality percentage was observed in case of treatment with Diacox combined with the UV protectant, where the corresponding mean mortality was 60%; whereas the lowest mean persistence expressed as zero mortality was occurred in case of spraying Tracer after 12 days of sunlight exposure. The biological activity of the tested compounds disappeared after 15 days for the tested compounds except Diacox mixed with Octyl palmitate, which recorded 27% mortality. A binomial generalized linear model (GLM) with a logit link function suggested significant difference in mortality percentage between the six compounds (Wald χ² (5) = 38.9, *p* < 0.001). Diacox + Octyl palmitate had the highest predicted mortality (75.7%) and was significantly the most effective compared to the other treatments (*p* < 0.001). There was no significant difference between Dipel + Octyl palmitate, Tracer + Octyl palmitate and Diacox as they showed moderate effect, but they represented significant difference when compared with Dipel and Tracer (Table 3). Finally, both Dipel and Tracer exhibited the lowest effect and there was no significant difference between each other (*p* = 0.396).

**Table 3:** Estimates of the binomial distribution model of the bioinsecticides used alone and combined with octyl palmitate against *S. littoralis*, 4th instar larvae under field-laboratory conditions.

Compounds used	B (Estimate)	Std. Error	Wald χ²	*P*-value	Predicted Mortality (%)
(Intercept)	− 0.57	0.20	8.20	0.004**	**–**
Dipel DF (Ref)	–	–	–	–	36
Dipel DF + Octyl palmitate	0.54	0.23	5.52	0.019*	49.5
Tracer	− 0.21	0.25	0.72	0.396	31
Tracer + Octyl palmitate	0.49	0.24	4.17	0.041*	48
Diacox	0.58	0.24	5.81	0.016*	50.7
Diacox + Octyl palmitate	1.54	0.27	32.62	0.000***	75.7

The synergistic ratios after combination between the UV protectant; Octyl palmitate with each Dipel DF, tracer and Diacox were 1.38, 1.55 and 1.49; respectively (Table 2). It was obvious that the ultraviolet protectant, Octyl palmitate markedly increased the persistence of the tested biocides.

Furthermore, the toxicity results of the three biocides either applied alone or mixed with the UV protectant, Tinuvin P were evaluated. Similar results were noticed, where the different applications of tested compounds revealed potent initial mortality at 0-day as well as their persistence after three days interval from their application. The initial mortality percentages ranged between 81 and 100% (Table 4). It was clear that the bioinsecticides persistence reduced after 12 days, as the mortality percentage of Dipel DF, Tracer and Diacox dropped to 3, 0 and 9. However, there were significant increase in mortality percentage (*P* < 0.05) to 20, 24 and 52 after mixed with Tinuvin P; respectively, which indicated the increase of persistence. On the other hand, the residue of the tested compounds disappeared completely after 15 days of application, except in case of both Tracer and Diacox mixed with the UV protectant, Tinuvin P, where the larval mortality was 9 and 32%; respectively. The GLM revealed a highly significant difference between all treatments (Wald χ² (5) = 39.87, *p* < 0.001). Tracer had the lowest predicted mortality (31%) and did not record a significant different with Dipel (*p* = 0.396). The highest mortality recorded (74.67%) by Diacox + Tinuvin P had a marked statistical significance in contrast to all other treatments (*p* < 0.001). Dipel + Tinuvin P, Tracer + Tinuvin P and Diacox produced moderate mortality but significantly greater compared to Dipel (*p* < 0.05) (Table 5).

**Table 4:** Potency of the ultraviolet protectant, Tinuvin P for increasing the toxicity of three bioinsecticides against the 4th instar larvae of *S. littoralis* under field-laboratory conditions.

Compounds used	Rate/plot	%Mean mortality ± SE						%Mean mortality	Improve ratio^*^	Toxicity Index	Relative potency levels
		Initial Kill 0-day	Days after treatment								
			3	6	9	12	15				
Dipel DF	10 g	84 ± 1.73^a^	72 ± 2.31^a^	42 ± 2.31^a^	15 ± 2.31^a^	3 ± 0.58^a^	0.00 ± 0.00^a^	36.0	–	48.21	1.16
Dipel DF + Tinuvin P	10 g + 1 g	84 ± 2.31^a^	80 ± 1.16^b^	72 ± 1.73^b^	40 ± 2.31^b^	20 ± 1.16^b^	0.00 ± 0.00^a^	49.33	1.37	66.06	1.59
Tracer	2.5 g	81 ± 1.16^a^	66 ± 1.73^a^	30 ± 1.16^c^	9 ± 0.00^a^	0.00 ± 0.00^a^	0.00 ± 0.00^a^	31.0	–	41.52	1.00
Tracer + Tinuvin P	2.5 ml + 1 g	81 ± 0.58^a^	81 ± 1.73^b^	72 ± 1.73^b^	51 ± 1.73^c^	24 ± 1.73^b^	9.00 ± 0.00^b^	53.03	1.71	70.98	1.71
Diacox	3 g	100 ± 0.00^b^	96 ± 2.31^c^	69 ± 2.89^b^	30 ± 1.16^d^	9 ± 0.58^c^	0.00 ± 0.00^a^	50.67	–	67.19	1.62
Diacox + Tinuvin P	3 g + 1 g	100 ± 0.00^b^	96 ± 2.31^c^	90 ± 1.73^d^	78 ± 3.46^e^	52 ± 2.31^d^	32 ± 1.16^c^	74.67	1.49	100	2.41

Different letters within the same column indicate statistical difference at the 0.05 level.

**Table 5:** Estimates of the binomial distribution model of the bioinsecticides used alone and combined with Tinuvin P against *S. littoralis*, 4th instar larvae under field- laboratory conditions.

Compounds used	B (Estimate)	Std. Error	Wald χ²	*P*-value	Predicted Mortality (%)
(Intercept)	− 0.57	0.20	8.20	0.004**	–
Dipel DF (Ref)	–	–	–	–	36
Dipel DF + Tinuvin P	0.53	0.23	5.30	0.021*	49.3
Tracer	− 0.21	0.25	0.72	0.396	31
Tracer + Tinuvin P	0.62	0.24	6.67	0.010**	53
Diacox	0.58	0.24	5.90	0.015*	50.7
Diacox + Tinuvin P	1.50	0.26	33.40	0.000***	74.7

Synergistic ratios resulted from combination between the UV protectant; Tinuvin P with each biocide, i.e., Dipel DF, Tracer and Diacox recorded 1.37, 1.71 and 1.49; respectively (Table 4).

The comparative toxicity of the three biocides, either used alone or in combination with the UV protectant UV-P was investigated. It was obvious that, application of tested compounds showed high initial mortalities at 0-day as well as their persistence after three days of treatment. After 6 days from spraying, the persistence of the tested compounds was declined moderately to cause larval mortality ranged between 30% and 81% (Table 6). There was a significant difference (*P* < 0.05) between treatments after the 12th day, as the residual effect of the insecticides were markedly decrease and become absent completely after 15 days, except for Diacox mixed with UV-P, which caused 12% mortality. The GLM model illustrated a marked significance between treatments (Wald χ² (5) = 27.6, *p* < 0.001) (Table 7). Treatment with Diacox + UV-P caused a higher significant when compared with the other compounds (*p* < 0.001). Additionally, Diacox showed greater significant difference in contrast to Dipel (*p* = 0.013) and Tracer (*p* = 0.006). However, Dipel, Dipel + UV-P, Tracer and Tracer + UV-P did not differ significantly (*p* > 0.05).

**Table 6:** Potency of the ultraviolet protectant, UV-P for increasing the toxicity of three bioinsecticides against the 4th instar larvae of *S. littoralis* under field-laboratory conditions.

Compounds used	Rate/plot	%Mean mortality ± SE						%Mean mortality	Improve ratio^*^	Toxicity Index	Relative potency levels
		Initial Kill 0-day	Days after treatment								
			3	6	9	12	15				
Dipel DF	10 g	84 ± 1.73^a^	72 ± 2.31^a^	42 ± 2.31^a^	15 ± 2.31^a^	3 ± 0.58^a^	0.00 ± 0.00^a^	36.00	–	55.24	1.16
Dipel DF + UV-P	10 g + 1 g	84 ± 1.73^a^	81 ± 3.46^b^	57 ± 1.73^b^	32 ± 1.16^b^	9 ± 0.58^b^	0.00 ± 0.00^a^	43.83	1.22	67.25	1.41
Tracer	2.5 ml	81 ± 1.16^a^	66 ± 1.73^a^	30 ± 1.16^c^	9 ± 0.00^c^	0.00 ± 0.00^c^	0.00 ± 0.00^a^	31.0	–	47.57	1.00
Tracer + UV-P	2.5 ml + 1 g	81 ± 1.73^a^	72 ± 1.73^a^	54 ± 1.73^b^	30 ± 1.73^b^	6 ± 1.16^d^	0.00 ± 0.00^a^	40.50	1.31	62.15	1.31
Diacox	3 g	100 ± 0.00^b^	96 ± 2.31^c^	69 ± 2.89^d^	30 ± 1.16^b^	9 ± 0.58^b^	0.00 ± 0.00^a^	50.67	–	76.98	1.62
Diacox + UV-P	3 g + 1 g	100 ± 0.00^b^	96 ± 2.31^c^	81 ± 1.73^e^	66 ± 1.73^d^	36 ± 1.73^e^	12 ± 0.58^b^	65.17	1.30	100	2.10

Different letters within the same column indicate statistical difference at the 0.05 level.

**Table 7:** Toxicity index and relative potency levels of the bioinsecticides used alone and combined with UV-P against *S. littoralis*, 4th instar larvae under field- laboratory conditions.

Compounds used	B (Estimate)	Std. Error	Wald χ²	*P*-value	Predicted Mortality (%)
(Intercept)	− 0.57	0.20	8.20	0.004**	–
Dipel DF (Ref)	–	–	–	–	36
Dipel DF + UV-P	0.32	0.22	2.10	0.147	43.8
Tracer	− 0.21	0.25	0.72	0.396	31
Tracer + UV-P	0.18	0.23	0.62	0.431	40.5
Diacox	0.60	0.24	6.18	0.013*	50.7
Diacox + UV-P	1.13	0.25	20.45	0.000***	65.2

Improvement ratios obtained from mixing the UV protectant; UV-P with the three biocides, Dipel DF, Tracer and Diacox exhibited 1.22, 1.31 and 1.30; respectively (Table 6). It could be concluded that the UV protectants played an important role in the improvement of the persistence of the three tested bioinsecticides.

### Comparison based on toxicity index and relative potency level values

**Sun**^14^ described the toxicity index as a method for comparing the relative toxicity of insecticides. According to this approach, the mean toxicity of the tested bioinsecticides, either used alone or combined with an UV protectant, against the 4th instar larvae of the *S*. *littoralis* (Tables 2, 4 and 6), was determined. In this investigation, the bioinsecticide Diacox, became the most promising when mixed with each UV protectant and was chosen as the standard toxicant, given an arbitrary index value of 100 units. The toxicity index values of Dipel DF, Dipel DF + Octyl palmitate, Tracer, Tracer + Octyl palmitate and Diacox were 47.57, 65.41. 40.97, 63.43 and 66.96%; respectively, when compared with the toxicity of Diacox + Octyl palmitate (Table 2). On the light of relative potency levels, Tracer showed the least toxic compound and was selected as the standard product for comparing the mean toxicity of the tested compounds. The toxicity levels of Dipel DF, Dipel DF + Octyl palmitate, Tracer + Octy lpalmitate, Diacox, and Diacox + Octyl pulmitate were 1.16, 1.60, 1.55, 1.63 and 2.44 times; respectively, relative to the toxicity of Tracer.

The role of the UV protectant, Tinuvin P in enhancing the toxicity and persistence of the tested biological agents was investigated. It was obvious that the toxicity index values of Dipel DF, Dipel DF + Tinuvin P, Tracer, Tracer + Tinuvin P and Diacox were 48.21, 66.06, 41.52, 70.98 and 67.19%; respectively, when compared with the toxicity of Diacox + Tinuvin P. According to relative potency levels, the mean toxicity values of Dipel DF, Dipel DF + Tinuvin P, Tracer + Tinuvin P, Diacox, and Diacox + Tinuvin P were 1.16,1.59, 1.71, 1.62 and 2.41 times higher; respectively, compared with the toxicity of Tracer against the tested insect (Table 4).

The influence of the UV protectant, UV-P on increasing the toxicity of the tested biological agents to control *S. littoralis* larvae was evaluated. It was clear that the toxicity index values of Dipel DF, Dipel DF + UV-P, Tracer, Tracer + UV-P and Diacox were 55.24, 67.25, 47.57, 62.15 and 76.98%, respectively; relative to the toxicity of Diacox + UV-P. Based on the relative potency levels, the mean toxicity values of Dipel DF, Dipel DF + UV-P, Tracer + UV-P, Diacox, and Diacox + UV-P were 1.16, 1.41, 1.31, 1.62 and 2.10 folds; respectively, compared with the toxicity of Tracer (Table 6).

### Quantitative analysis of protein patterns of treated and untreated larvae of *S. littoralis* by using electrophoretic profiles

**Fig. 1:**
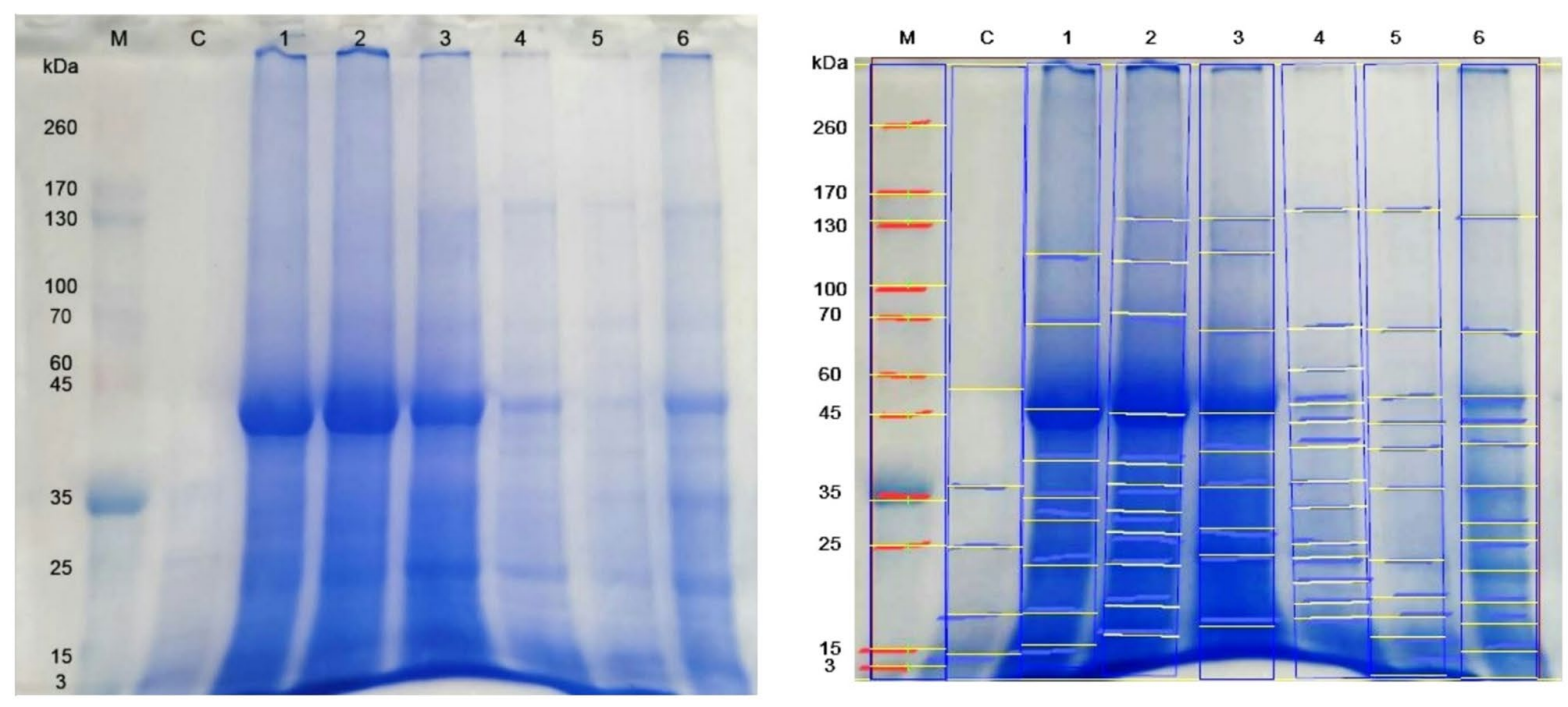
SDS-PAGE analysis of control and treated samples of *S. littoralis* larvae. M: Protein marker C: Samples of control. 1: Samples of Dipel DF. 2: Samples of Dipel DF + Octyl palmitate. 3: Samples of Dipel DF + Tinuvin P. 4: Samples of Dipel DF + UV-P. 5: Samples of Tracer. 6: Samples of Tracer + Octyl palmitate.

**Fig. 2:**
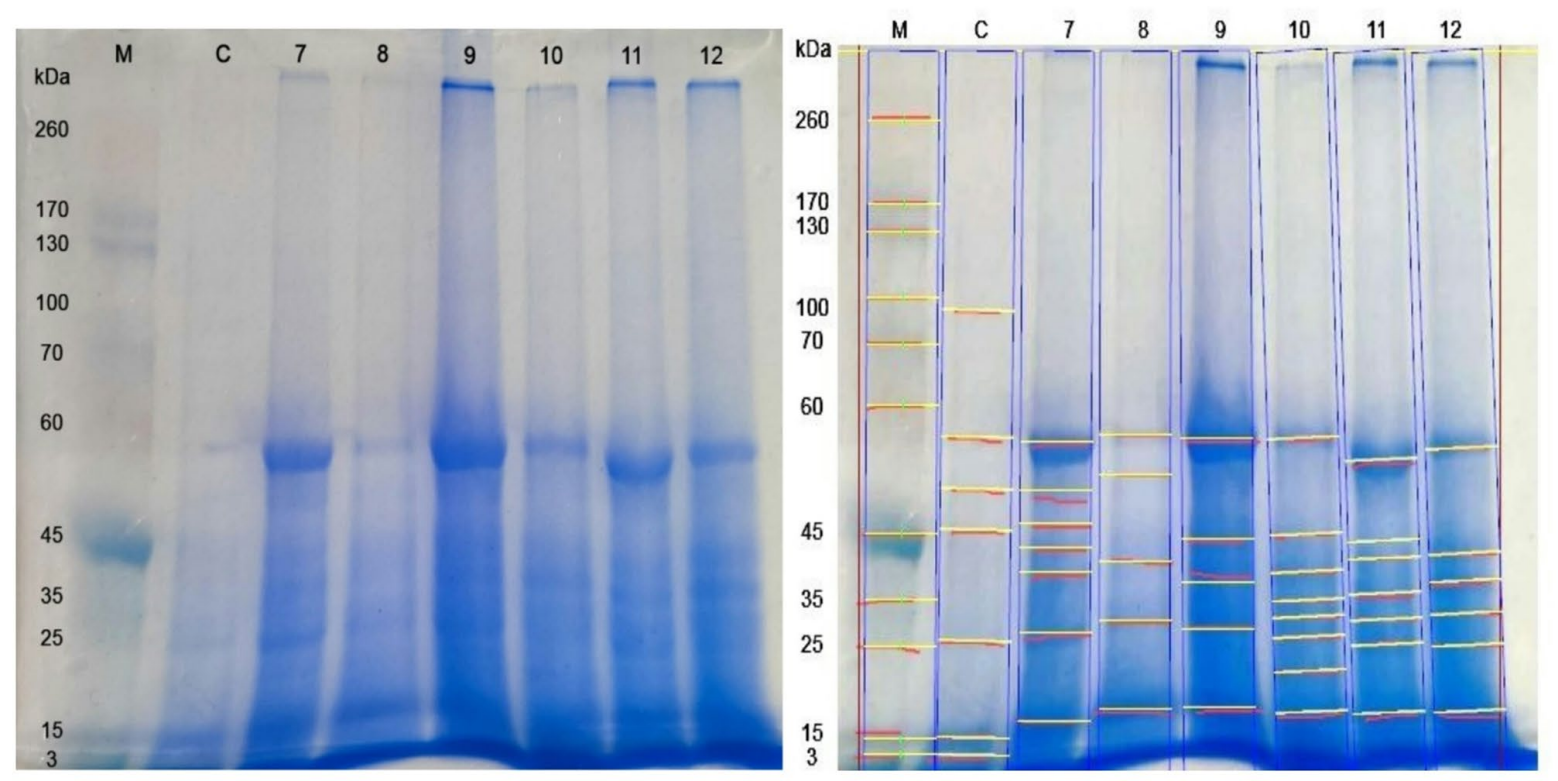
SDS-PAGE analysis of control and treated samples of *S. littoralis* larvae. M: Protein marker C: Samples of control 7: Samples of Tracer + Tinuvin P. 8: Samples of Tracer + UV-P 9: Samples of Diacox. 10: Samples of Diacox + Octylpulmitate 11: Samples of Diacox + Tinuvin P. 12: Samples of Diacox + UV-P.

Electrophoretic protein pattern of the 4th larval instar of untreated *S. littoralis* and treated with the three biological agents alone and after adding the different UV protectants showed differences in the numbers of protein fragments. According to their molecular weight values, the samples were separated into 25 different bands (Figs. 1 and 2).

The total number of protein bands in control samples was 10 with molecular weights ranged between 2.74 and 90.47 kDa (Tables 8 and 9). While the number of bands in treated larvae with the three biological agents; Dipel DF, Tracer and Diacox were 9, 11, 5 bands with molecular weights ranged between (15.32-113.61), (1.34-145.48) and (17.84–55.68) kDa; respectively. The addition of Octyl palmitate protectant to Dipel DF, Tracer and Diacox led to an increase in the total bands to 11, 13 and 8 bands with molecular weights ranged from (16.06-134.49), (1.05-136.93) and (17.36–55.78) kDa; respectively. Mixing Tinuvin P protectant with Dipel DF, Tracer and Diacox, produced 9, 7 and 7 protein bands with molecular weight ranges of (16.83-134.58), (16.51–55.37) and (17.28–53.14) kDa; respectively. In the final combination, UV-P protectant with Dipel DF, Tracer and Diacox yielded 13, 5 and 6 bands with molecular weights varying between (17.56-145.48), (17.68–56.20) and (17.44–54.55) kDa; respectively.

**Table 8:** Molecular weight of SDS-protein patterns of both treated and control sample of *S. littoralis* larvae.

Rows	Molecular weight of bands							
	Marker	Control	Dipel DF	Dipel DF + Octyl palmitate	Dipel DF + Tinuvin *P*	Dipel DF + UV-*P*	Tracer	Tracer + Octyl palmitate
r1	260	–	–	–	–	–	–	–
r2	170	–	–	–	–	–	–	–
r3	–	–	–	–	–	145.48	145.48	–
r4	130	–	–	134.49	134.58	–	–	136.93
r5	–	–	113.61	110.05	114.42	–	–	–
r6	100	90.47	–	–	–	–	–	–
r7	70	–	–	73.36	–	–	–	–
r8	–	–	68.86	–	67.75	68.07	67.91	67.28
r9	–	–	–	–	–	–	–	–
r10	60	–	–	–	–	60.99	–	–
r11	–	54.29	–	–	–	–	–	–
r12	–	49.78	–	–	–	48.81	51	51.64
r13	45	45.42	47.01	45.28	45.57	44.10	43.76	43.54
r14	–	–	39.34	38.94	40.35	40.97	40.76	41.28
r15	–	–	–	–	–	–	–	–
r16	35	36.54	35.18	36.64	36.36	37.01	36.27	36.45
r17	–	–	30.25	32.23	–	33.27	–	29.49
r18	25	25.92	–	27.50	28.38	25.48	–	25.97
r19	–	–	22.67	22.77	23.96	23.65	23.35	22.10
r20	–	–	–	–	–	20.91	–	–
r21	–	17.94	17.94	18.56	–	18.88	19.36	18.88
r22	–	–	–	16.06	16.83	17.56	17.63	17.04
r23	15	15.14	15.32	–	–	–	15.92	–
r24	–	10.03	–	–	–	–	–	11.78
r25	3	2.74	–	–	–	–	1.34	1.05

**Table 9:** Molecular weight of SDS-protein patterns of both treated and control sample of *S. littoralis* larvae.

Rows	Molecular weight of bands							
	Marker	Control	Tracer + Tinuvin *P*	Tracer + UV-*P*	Diacox	Diacox + Octyl palmitate	Diacox + Tinuvin *P*	Diacox + UV-*P*
r1	260	–	–	–	–	–	–	–
r2	170	–	–	–	–	–	–	–
r3	–	–	–	–	–	–	–	–
r4	130	–	–	–	–	–	–	–
r5	–	–	–	–	–	–	–	–
r6	100	90.47	–	–	–	–	–	–
r7	70	–	–	–	–	–	–	–
r8	–	–	–	–	–	–	–	–
r9	–	–	–	–	–	–	–	–
r10	60	–	–	–	–	–	–	–
r11		54.29	55.37	56.20	55.68	55.78	53.14	54.55
r12	–	49.78	49.68	51.38	–	–	–	–
r13	45	45.42	46.02	–	44.14	45	43.85	–
r14	–	–	42.60	40.46	–	–	40.99	41.65
r15	–	–	38.80	–	37.33	38.93	–	37.45
r16	35	36.54	–	–	–	34.79	35.80	–
r17	–	–	–	29.94	–	30.85	30.30	31.41
r18	25	25.92	27.36	–	28.36	26.71	25.15	25
r19	–	–	–	–	–	–	–	–
r20	–	–	–	–	–	21.70	–	–
r21	–	17.94	–	17.68	17.84	17.36	17.28	17.44
r22	–	–	16.51	–	–	–	–	–
r23	15	15.14	–	–	–	–	–	–
r24	–	10.03	–	–	–	–	–	–
r25	3	2.74	–	–	–	–	–	–

The bands 13 and 16 with molecular weights 35 and 45 kDa were detected only in the control group and in larvae treated with Dipel DF, Dipel DF + Octyl palmitate, Dipel DF + Tinuvin P and Dipel DF + UV-P. Another distinct band 21 (17.94 kDa) was present only in untreated larvae and those exposed to Diacox, Diacox + Octyl palmitate, Diacox + Tinuvin P and Diacox + UV-P.

One characteristic band 6 (90.47 kDa) appeared in untreated sample, whereas the band 7 was unique to larvae treated with Dipel DF + Octyl palmitate with molecular weight 73.36 kDa. Additionally, the band 10 (60.99 kDa) was specific to larvae treated with Dipel DF + UV-P.

Relative to untreated sample, exposure to the three biological agents; Dipel DF, Tracer and Diacox signified the appearance of 5, 5 and 1 abnormal bands; respectively. Those bands were present at rows 5, 8, 14, 17 and 19 (113.61, 68.86, 39.34, 30.25 and 22.67 kDa), rows 3, 8, 14, 19 and 22 (145.48, 67.91, 40.76, 23.35 and 17.63 kDa) and row 15 (37.33 kDa); respectively. Conversely, disappearance of 6, 4 and 6 normal bands following treatment with Dipel DF, Tracer and Diacox from the rows 6, 11, 12, 18, 24 and 25, rows 6, 11, 18 and 24 and rows 6, 12, 16, 23, 24 and 25; respectively.

Adding of Octyl palmitate protectant to Dipel DF, Tracer and Diacox, led to the emergence of 7, 6 and 3 abnormal bands at rows 4, 5, 7, 14, 17, 19 and 22 (134.49, 110.05, 73.36, 38.94, 32.23, 22.77 and 16.06 kDa), rows 4, 8, 14, 17, 19 and 22 (136.93, 67.28, 41.28, 29.49, 22.10 and 17.04 kDa) and rows 15, 17 and 20 (38.93, 30.85 and 21.70); respectively. Alternatively, number of normal protein bands (7, 3 and 5 bands) disappeared at rows 6, 11, 12, 18, 23, 24 and 25, rows 6, 11 and 23 and rows 6, 12, 23, 24 and 25; respectively.

Mixing Tinuvin P protectant with Dipel DF, Tracer and Diacox, resulted in the emergence of 6, 3 and 2 abnormal bands at rows 4, 5, 8, 14, 19 and 22 (134.58, 114.42, 67.75, 40.35, 23.96 and 16.83 kDa), rows 14, 15 and 22 (42.60, 38.80 and 16.51 kDa), rows 14 and 17 (40.99 and 30.30); respectively. By contrast, normal protein band 7, 6 and 5 disappeared from the rows 6, 11, 12, 21, 23, 24 and 25, rows 6, 16, 21, 23, 24 and 25 and rows 6, 12, 23, 24 and 25; respectively.

Combination of UV-P protectant with Dipel DF, Tracer and Diacox, led to the presence of 8, 2 and 3 abnormal bands at rows 3, 8, 10, 14, 17, 19 20 and 22 (145.48, 68.07, 60.99, 40.97, 33.27, 23.65, 20.91 and 17.56 kDa), rows 14 and 17 (40.46 and 29.94 kDa), and rows 14, 15 and 17 (41.65, 37.45 and 31.41); respectively. On the other hand, 5, 7 and 7 normal bands disappeared from rows 6, 11, 23, 24 and 25, rows 6, 13, 16, 18, 23, 24 and 25 and rows 6, 12, 13, 16, 23, 24 and 25; respectively.

## Discussion

It is known that biocides including Emamectin benzoate, Spinosad and *Bacillus thuringiensis* (*Bt.*) are less stable and gradually degrade by time under environmental conditions like high temperatures, sunlight and ultraviolet (UV) radiation^16–18^. It is important to improve the efficacy of these biological agents to overcome their limited application against foliar pests in the field due to their sensitivity to high temperatures and UV radiation. Research has shown that the addition of three ultraviolet protectants namely, Octylpulmitate, Tinuvin P and UV-P to some biological control agents can improve their efficacy to control the 4th larval instar of the cotton leaf worm *Spodoptera littoralis* (Boisd.). Therefore, UV protectants are promising materials that can increase the persistence of biocides under field conditions. The generated data revealed that Emamectin benzoate demonstrated the highest toxicity, followed by *Bt.*, while Spinosad recorded the lowest effect.

In the same manner, **El-Saleh et al.**^19^ assessed the efficacy of five insecticides; Chlorpyrifos, Lufenuron, Cypermethrin, Emamectin benzoate, and Spinosad against *S. littoralis* larvae under laboratory and field conditions. The results revealed that Emamectin benzoate was the most effective among the tested insecticides, while Spinosad exhibited the least level of toxicity. **Salem et al.**^20^ controlled 4th instar larvae of *S. littoralis* using Emamectin benzoate, Spinosad and Dinotefuran and compared their toxicity based on LC_50_ values which were 0.8, 201.7 and 3979.6 ppm; respectively.

Mortality of 4th instar larvae of *S. littoralis* was higher after combination of Octylpulmitate, Tinuvin P and UV- P with the three biological agents; Dipel DF, Tracer and Diacox. Referring to the mean mortality percentages of *S. littoralis*, 4th instar larvae, after 15 days of treatments, the results indicated that the mean mortality percentages increased from 36.0, 31.0 and 50.67% after treatment with the bioinsecticides; Dipel DF, Tracer and Diacox alone to (49.50, 48.0 and 75.67%), (49.33, 53.03 and 74.67%) and (43.83, 40.5 and 65.17%) after addition of Octylpalmitate, Tinuvin P and UV- P; respectively to the bioinsecticides. From the experimental findings, Diacox + UV protectants exhibited the highest toxicity to control 4th larval instar of *S. littoralis* compared to the other treatments. Dipel + UV protectants, Tracer + UV protectants and Diacox showed moderate effect, while both of Dipel and Tracer showed the lowest effect. Similarly, **Acar and Sipes**^21^ found that the mortality of mealworm larvae *Tenebrio molitor* increased from 5% to reach over 90% after protection of *Steinernema feltiae* nematodes with the UV protectant; Para-amino benzoic acid (PABA) after 12 h of UV radiation exposure. The results agreed with **Hadapad et al.**^11^ who revealed that the larvicidal activity of *Bacillus sphaericus* ISPC-8 spores was reduced to 57.7% and 43% after UV-B exposure for 6 and 20 h and completely lost after 24 h. Whereas, more than 87% of the larvicidal activity remained after UV-B exposure for 168 h, after addition of UV-Protectants like PABA and Congo red to *Bacillus sphaericus* ISPC-8 spores. **Sukirno et al.**^22^ used sericin extracted from cocoon of the eri silkworm, *Samia ricini* and atlas moth, *Attacus atlas* as UV-B protectants for *Bacillus thuringiensis* to control the larvae of tobacco cutworm, *Spodoptera litura*. The mortality percentages of tobacco cutworm increased from 46.67% in *Bt*. + H_2_O treatment samples to 75% and 81.67% after addition of atlas and eri sericin to *Bt*.

Furthermore, **El-Husseini et al.**^10^ tested four natural products; black tea extract, clay, starch and glycerin as UV protectant for enhancement of *S. littoralis* nucleopolyhedrovirus (*Sl*NPV) efficiency to control *S. littoralis* larvae under field conditions. The results proved the usage of black tea extract and clay as UV protectant at 2.5, 5 and 10% were more suitable than either glycerin or starch. Also, the obtained results were supported with those published by **Kaiser et al.**^23^ who revealed that the addition of natural UV protectant can increase the persistence and efficacy of entomopathogenic fungi *Beauvaria bassiana* spores under field conditions.

Changes in protein structure may result due to the balance among degradation of functional and structural nutrients during ontogeny as well as reflect response of some physiological conditions^24^. The SDS-PAGE of protein that extracted from treated and untreated 4th instar larvae of *S. littoralis*, indicated differences in the numbers and molecular weight of protein fragments. Comparing with untreated larvae; all treated samples either with biological agents alone or combined with the three ultraviolet protectants have caused detection of abnormal new bands and absence of some normal bands. SDS-PAGE analysis studied by **Hadapad et al.**^11^ confirmed a similar response after addition of two UV-Protectant; PABA and congo red to *Bacillus sphaericus* ISPC-8 spores resulted in presence of toxin bands (41.9 kDa and 51.4 kDa) of *B. sphaericus* ISPC-8 after exposure to UV-B radiation for 168 h and disappearance of these binary toxin bands of unprotected *B. sphaericus* after UV-B radiation exposure for 24 h. Instability of 41.9 kDa and 51.4 kDa toxic proteins of *B. sphaericus* may be caused by the generation of peroxide radicals from UV radiation, which may be connected to the degradation of toxin protein bands which was reflected in loss of *B. sphaericus* activity to control larvae of *Culex quinquefasciatus*. **Li et al.**^25^ found that exposure of red and green pea aphids *Acyrthosiphon pisum* to solar ultraviolet (UV-B radiation) caused a significant decrease in the protein percentage content in treated green and red aphids by 8.77% -33.69% and 12.56%-25.70% in the treated green aphids. However, in control, the protein content of aphids remained stable. As the long-term UV-B radiation exposure resulted in protein transportation and protein synthesis inhibition.

The comparison between, SDS PAGE results, of untreated larvae and those treated with Dipel, and Dipel + UV protectants demonstrated the appearance of abnormal new bands at ranges between 65 and 73 kDa after addition of Octyl palmitate, Tinuvin P, and UV-P. These multiple bands that may be corresponding to secondary bands of the δ-endotoxins Cry2Aa and Cry11Aa^26,27^. Also, new multiple bands detected after exposure to Dipel + Octyl palmitate and Dipel + Tinuvin P with range between 130 and 135 kDa and 27–29 kDa, which could be major and minor low molecular bands; respectively, of the δ-endotoxins Cry1Aa, Cry1Ab, and Cry1Ac^28^. Using Dipel + UV-P as well as in absence of UV protectant (Dipel only), the major or minor bands disappeared which may reflect their lower mortality percentage compared with Dipel + Octyl palmitate and Dipel + Tinuvin P. Similar results obtained by **Jallouli et al.**^29^ who noticed a decline of delta-endotoxin bands after 3 days by using molasses as UV protectant. They suggested that UV radiations induced peroxide radicals formation causing instability of the 65–70 kDa and 130 kDa delta-endotoxin bands.

Closely related findings obtained by **Abd El Mageed et al.**^30^ who detected 11 protein bands after treatment with Spinosad against *S*. *littoralis*, whereas 9 bands were identified in control. They explained that the insects may use this novel band to detoxify the insecticides they were exposed to. Similar results noticed by **Negm et al.**^31^ who recorded alterations including the disappearance of some normal protein bands and appearance of new abnormal bands after treatment with Spinosad combined with sesame and lemongrass oils and their mixture to control the peach fruit fly, *Bactrocera zonata*. They claimed that an increase in protein synthesis could be the result of a new protein band’s formation, whereas the absence of other could be linked to their disintegration. Despite the ability of UV protectants to slow down biological agent degradation, they may lose some of their protective properties over time as a result of weathering, irrigation, or plant development. Therefore, more studies representing long-term applications under different environmental conditions are needed to carry out. Also, not all biological control agent formulation may compatible with UV protectants. Chemical interactions in some cases may occasionally lower insecticidal activity or microbiological viability. Residues of some synthetic UV protectants may interact with non-target organisms and beneficial insects. Furthermore, clarification is also needed about the effect on natural enemies.

## Conclusion

The present study highlighted the critical role of UV-protectants, Octylpalmitate, Tinuvin P and UV-P in maintaining the efficacy and residual activity of bioinsecticides, Dipel 6.4% DF, Tracer and Diacox, to control the Egyptian cotton leaf worm, *Spodoptera littoralis* under field conditions. Among the tested formulations, Diacox + UV protectant provided the highest residual activity, while Tracer exhibited the lowest persistence, with zero mortality after 12 days. By 15 days, most bioinsecticide treatments lost efficacy, except when Diacox combined with Octyl palmitate, Tinuvin P, and UV-P, which caused 12–32% mortality. Furthermore, SDS-PAGE analysis revealed that treatments induced changes in *S. littoralis* protein profiles, including the appearance of new protein bands and disappearance of normal bands. This reflect that there were some physiological stress reactions happened as a result of the combined treatments. However, the studies may not represent long-term applications and were carried out under particular field conditions. More researches across different seasons and environments before widespread use is advised.

## Data Availability

The datasets used and/or analyzed during the current study are available from the corresponding author upon reasonable request.

## References

[1] Abdel-Aziz, H. & El-Gohary, E. E. Biochemical studies on cotton leaf worm, *Spodoptera littoralis* (Boisd.) treat with three novel insecticides. 39,77–90. *Bull Ent Soc. Egypt. Econ. Ser* (2013).

[2] Ishaaya, I., Kontsedalov, S. & Horowitz, A. R. Emamectin, a novel insecticide for controlling field crop pests. *Pest Manag Sci.* **58** (11), 1091–1095. 10.1002/ps.535 (2002). 12449526 10.1002/ps.535

[3] Al- Ashry, H. A. Potency of some biocontrol agents in suppressing the population of the American bollworm, *Helicoverpa armigera* (Hub.). Ph. D. Thesis, Institute of Environmental Studies and Research, Ain -Shams Univ. (PP 139). (2018).

[4] Thompson, G. D. & Sparks, T. C. Spinosad: a green natural product for insect control. *In*: Advancing Sustainability Through Green Chemistry and Engineering, Vol. 823, 61–73. American Chemical Society, Washington, DC. 10.1021/bk-2002-0823.ch005 (2002).

[5] Abdel- Mageed, A. E. M. Fate of spinosad under Egyptian environment conditions. *Egypt. J. Agric. Res.* **2** (2), 265–267 (2005).

[6] Krieg, A., Groner, A., Huber, J. & Matter, M. The effect of medium- and long-wave ultraviolet rays (UV-B and UV-A) on insect-pathogenic bacteria and viruses and their influence by UV-protectants. *Nachr. Bl Dtsch. Pflanzenschutzd*. **32** (7), 100–106 (1981).

[7] Jeyakuma, P. & Gupta, G. P. Impact of UV and white lights on the bio-potency of *Bacillus Thuringiensis* against *Helicoverpa armigera* hubner. *Ann Plant Sci.* **7** (2), 121–124 (1999).

[8] Kucuk, S. D., Gerengi, H. & Guner, Y. The effect of Tinuvin derivatives as an ultraviolet (UV) stabilizer on EPDM rubber. *Period eng. nat. sci.* **6** (1), 52–62. 10.21533/pen.v6i1.157 (2018).

[9] Fiume, M. M. et al. Safety assessment of alkyl esters as used in cosmetics. *Int. J. Toxicol.* **34** (Suppl 2), 5S–69S. 10.1177/1091581815594027 (2015). 26362120 10.1177/1091581815594027

[10] El-Husseini, M. M., El-Khaleil, A. D., El-Aw, M. A. & Kasem, A. Testing some natural additives as protectants for *S. littoralis* nucleopolyhedro virus from sunlight inactivation. *Egypt. J. Biol. Pest Cont.* **22** (2), 191–196 (2012).

[11] Hadapad, A. B., Hire, R. S., Vijayalakshmi, N. & Dongre, T. K. UV-Protectants for the biopesticide based on *Bacillus sphaericus* Neide and their role in protecting the binary toxins from UV radiation. *J. Invertebr Pathol.* **100** (3), 147–152. 10.1016/j.jip.2008.12.003 (2009). 19167401 10.1016/j.jip.2008.12.003

[12] Khidr, A. A., Sayed, M. & Al-Ashry, H. A. A. Evaluating the efficiency of photo active compounds against armyworm, *Spodoptera Frugiperda* (Lepidoptera: Noctuidae). *Egypt. J. Plant. Protec Res. Inst.* **5** (4), 370–381 (2022).

[13] Abbott, W. S. A. Method of computing the effectiveness of an insecticide. *J. Econ. Etomol*. **18** (2), 265–267. 10.1093/JEE/18.2.265A (1925).

[14] Sun, Y. P. Toxicity index-An improved method of comparing the relative toxicity of insecticides. *J. Econ. Entomol.* **43** (1), 45–53. 10.1093/jee/43.1.45 (1950).

[15] Laemmli, U. K. Cleavage of structural proteins during the assembly of the head of bacteriophage T4. *Nature* **227**, 680–685. 10.1038/227680a0 (1970). 5432063 10.1038/227680a0

[16] Gupta, S., Dikshit, A. K. & Biopesticides An ecofriendly approach for pest control. *J. Biopesticides*. **3** (1), 186–188 (2010).

[17] Villaverde, J. J., Sevilla-Moran, B., Sandin-Espana, P., Lopez-Goti, C. & Alonso-Prados, J. L. Biopesticides in the framework of the European pesticide regulation (EC) 1107/2009. *Pest Manage. Sci.* **70**, 2–5. 10.1002/ps.3663 (2014). 10.1002/ps.3663 24174346

[18] Jalali, E., Maghsoudi, S. & Enhancing UV radiation protection of Bacillus Thuringiensis formulations using sulfur quantum dots: synthesis and efficacy evaluation. *Sci. Rep.* **14** (1), 17384. 10.1038/s41598-024-68595-1 (2024). 39075143 10.1038/s41598-024-68595-1 PMC11286829

[19] El-Saleh, M. A. et al. Comparative toxicological effects of insecticides and their mixtures on *Spodoptera littoralis* (Lepidoptera: Noctuidae). *Insects* **16** (8), 821. 10.3390/insects16080821 (2025). 40870622 10.3390/insects16080821 PMC12386461

[20] Salem, R. R., Saleh, A. A., Elgohary, L. R. & Hamada, M. S. The toxicity and biochemical activity of Spinosad, Emamectin benzoate and Dinotefuran on *Spodoptera littoralis* (Boisd). *J. Plant. Prot. Pathol.* **15** (9), 293–297. 10.21608/jppp.2024.317030.1259 (2024).

[21] Acar, I. & Sipes, B. Enhancing the biological control potential of *Steinernema Feltiae* with protection from desiccation and UV radiation. *Biol. Control*. **169**, 104874. 10.1016/j.biocontrol.2022.104874 (2022).

[22] Sukirno, S. et al. The effectiveness of *Samia Ricini* drury (Lepidoptera: Saturniidae) and *Attacus atlas* L. (Lepidoptera: Saturniidae) cocoon extracts as ultraviolet protectants of *Bacillus Thuringiensis* for controlling *Spodoptera Litura* Fab. (Lepidoptera: Noctuidae). *Int. J. Trop. Insect Sci.* **42** (1), 255–260. 10.1007/s42690-021-00540-5 (2022).

[23] Kaiser, D., Bacher, S., Mène-Saffrané, L. & Grabenweger, G. Efficiency of natural substances to protect *Beauveria Bassiana* conidia from UV radiation. *Pest Manag Sci.* **75** (2), 556–563. 10.1002/ps.5209 (2019). 30221461 10.1002/ps.5209 PMC6587961

[24] Attia, R. G. M. Evaluation of a plant essential oil encapsulated with silica nano particles against the rice moth, *Corcyra cephalonica* Stainton (Lapidoptera: Pyralidae). Ph. D. Thesis, Faculty of science, Ain shams Univ. (2020).

[25] Li, C. et al. The impact of ultraviolet-B radiation on the sugar contents and protective enzymes in *Acyrthosiphon Pisum*. *Insects* **12** (12), 1053. 10.3390/insects12121053 (2021). 34940141 10.3390/insects12121053 PMC8708437

[26] Gothandaraman, R., Venkatasamy, B., Thangavel, T., Eswaran, K. & Subbarayalu, M. Molecular characterization and toxicity evaluation of Indigenous Bacillus Thuringiensis isolates against key lepidopteran insect pests. *Egypt. J. Biol. Pest Control*. **32** (1), 143. 10.1186/s41938-022-00639-y (2022).

[27] Lai, L. et al. *Bacillus Thuringiensis* cyt proteins as enablers of activity of cry and Tpp toxins against *Aedes albopictus*. *Toxins* **15** (3), 211. 10.3390/toxins15030211 (2023). 36977103 10.3390/toxins15030211 PMC10054650

[28] Reyaz, A. L., Gunapriya, L. & Indra Arulselvi, P. Molecular characterization of Indigenous *Bacillus Thuringiensis* strains isolated from Kashmir Valley. *3 Biotech.* **7** (2), 143. 10.1007/s13205-017-0756-z (2017). 28597156 10.1007/s13205-017-0756-z PMC5465046

[29] Jallouli, W., Sellami, S., Sellami, M. & Tounsi, S. Efficacy of Olive mill wastewater for protecting *Bacillus Thuringiensis* formulation from UV radiations. *Acta Trop.* **140**, 19–25. 10.1016/j.actatropica.2014.07.016 (2014). 25093915 10.1016/j.actatropica.2014.07.016

[30] Abd, E. et al. Biochemical and molecular characterization studies of Spinosad, Methoxyfenozide and extrem on the cotton Leafworm, spodoptera littoralis (Boisd). *Egypt. Acad. J. Biol. Sci. Entomol.* **15** (1), 25–32. 10.21608/EAJBSA.2022.219047 (2022).

[31] Negm, A. A. K. H., Elsayed, D. A. E., Shafei, E., Mosallam, A. M., Maamoun, S. A. & A. M. Z., & M. Synergistic activity of Lemongrass and Sesame oils on spinosad: A new approach to control the Peach fruit fly; *Bactrocera zonata* (Saunders, 1841), referring to their effect on the adult biological and protein aspects. *Pol. J. Entomol.* **91** (2), 84–93. 10.5604/01.3001.0015.9179 (2022).

